# COVID-19, SARS-CoV-2, and Export Controls

**DOI:** 10.1089/hs.2020.0048

**Published:** 2020-08-19

**Authors:** Piers Millett, Paul Rutten

**Affiliations:** Piers Millett, PhD, is Vice President for Safety and Security, iGEM Foundation, Boston, MA; and a Senior Research Fellow, Future of Humanity Institute, University of Oxford, Oxford, UK. Paul Rutten, MRes, is a PhD Student, Department of Plant Sciences, University of Oxford, Oxford, UK.

**Keywords:** COVID-19, SARS, Dual-use science, International coordination, Export controls

## Abstract

Export controls are intended to prevent the proliferation of materials that could be misused to make biological weapons. They are not intended to stifle critical research and development in the midst of a pandemic. This article explores how and why export controls might apply to severe acute respiratory syndrome coronavirus 2, the virus that causes coronavirus disease 2019. It outlines the taxonomic and genetic factors associated with the current approach to export control lists and discusses how they lead to unnecessary ambiguity. The authors describe ways in which the current export control systems might be revised in the short, medium, and long term, including sequence, disease, and function-based approaches.

## Introduction

In the midst of major social disruption caused by the current coronavirus disease 2019 (COVID-19) pandemic, now may seem like a strange time to be thinking about the minutiae of international arms control and disarmament infrastructure. However, when the measures we created to prevent disease from being used as a weapon become barriers to critical research during a pandemic, revisiting export controls becomes timely and important.

To understand how severe acute respiratory syndrome coronavirus 2 (SARS-CoV-2)—the virus responsible for COVID-19—works and which public health interventions may be warranted, access to samples of the virus or its fragments is required. Access to the virus is also vital for testing the efficacy of surveillance and therapeutics, developing a vaccine, and conducting other studies.

## The Public Health Prerogative for Access

Developing medical and policy interventions depends on rapid, reliable, and robust research and development. Today, much of this research requires moving clinical and viral samples—such as fragments of SARS-CoV-2 genetic material—around the world. Efforts that rely on these samples include identifying where and in whom the virus is present and detecting antibodies that show who has been exposed to the disease. Samples are also used as targets for drugs being developed to treat the disease or prevent its spread. In the case of SARS-CoV-2 genetic material fragments, most are commercially synthesized and shipped internationally to where the research is taking place. To that end, the commercial gene synthesis industry is playing an important role in efforts to manage and mitigate this global public health emergency.^1^

## Security Sanctions that Control Access

To reduce the risk of diseases being developed into weapons, many countries control access to—and especially export of—certain dangerous pathogens. Export controls typically focus on materials that are not widely available internationally. They usually apply to isolates of the pathogens, not to clinical samples. Controls can also cover components of these pathogens and dual-use equipment that can be used to work with them.

Notable efforts have been made in recent decades to review and revise export controls in light of sociopolitical and technical developments. Some efforts have focused on updating export controls on a larger scale to better reflect post-Cold War international relations and to help resolve competing requirements for security and competitive advantage.^2^ Others have focused specifically on export controls for biological agents, such as the possible role of restricting access to dual-use information.^3^ Many of the shortcomings of current approaches discussed in the following sections have been described before, such as the challenges of using list-based and sequence-based approaches.^4^ Here, we consider these challenges in light of the ongoing pandemic and as they specifically relate to the causative pathogen.

Export controls help prevent those intent on causing harm from accessing the critical materials most likely to be used as part of a biological weapons program. Whether this includes SARS-CoV-2 remains a question. If the virus could be used as a suitable weapon, it should be covered by the export controls intended to prevent that misuse, assuming it meets other relevant criteria such as not being widely available. If the virus would not make a suitable weapon, it should not be covered by control measures. In either case, there should be clarity as to whether export controls apply to SARS-CoV-2 or not.

The need to reduce the likelihood of biological weapons development does not disappear during a pandemic. Having measures in place to prevent deliberate misuse becomes even more important when its potential impacts are so visible and raw materials are so readily available. However, the current widespread availability of SARS-CoV-2 could also mean that export controls no longer apply to the virus or, at minimum, are rendered moot.

It is important to examine what happens when export controls are applied to a virus causing a pandemic. Any export controls that impede the ability to do research on SARS-CoV-2 during the current pandemic are inappropriate and harmful. We have described elsewhere how time-consuming the procedures can be to obtain a license for the export of listed microbes and goods.^5^ In some cases, even determining whether a license is required can take months. Ideally, no controls should be placed on international transfers of SARS-CoV-2 samples or related genetic sequences, at least during the current pandemic. At a minimum, a fast track should be required for any license requests associated with SARS-CoV-2. In a more typical scenario, long delays could be factored into research timeframes, but adhering to such rules in the midst of a worldwide pandemic is more challenging and can be measured not just in dollars but in lives lost. What needs to be determined then is whether researchers are currently applying for export licenses to move the virus or its genes internationally, and what happens when much of the infrastructure and capacity of governments has been focused on dealing with pressing public health needs rather than export license requests.

## Export Controls Applied to SARS-CoV-2

Whether the SARS-CoV-2 virus is covered by export controls remains a point of confusion around the world. The US Department of Commerce Bureau of Industry and Security has released guidance that SARS-CoV-2 is not currently subject to these measures.^6^ Similar guidance from other countries is less visible. For example, since the beginning of the pandemic the United Kingdom has updated the following guidelines: *Consolidated List of Strategic Military and Dual-Use Items that Require Export Authorisation*^7^ in January 2020, notices to exporters^8^ in April (and again in July) 2020, and UK strategic export control lists^9^ in April 2020. However, none of these updates, nor the website of the Export Control Joint Unit of the Department for International Trade, provided any guidance on the export of SARS-CoV-2.^10^ When contacted, relevant authorities confirmed they had “already discussed the classification of the current virus outbreak, SARS-CoV-2, and concluded this virus is not listed in UK strategic export control lists” (J. King, UK Department for International Trade, written communication, April 15, 2020). They also indicated that they were “advising exporters that licenses are not required to export samples of SARS-CoV-2 or its genetic elements,” with the caveat common to many export control regimes that exporters should still block transfers where they “know or suspect the export is in relation to use in a WMD [weapons of mass destruction] program.” How or when such advice is given was not discussed.

Similarly, the Australia Group, an international forum for harmonizing biological and chemical-related export controls, has been silent on whether this virus requires an export license.^*^ An intersessional meeting of the Australia Group occurred in February 2020 as knowledge of the virus's spread was becoming more well known. Yet, the only output from that meeting was to add Novichok nerve agent precursors to the list of chemical precursors.^11^ Other than the United States, it remains unclear whether export of the virus requires a license from relevant governments.

## Application of Existing Export Controls

Existing export controls may already apply to SARS-CoV-2 for the reasons described in this section. The first reason is the way in which controlled viruses are described in export control regulations. Unfortunately, most export control lists remain focused on taxonomy—listing only the names of the pathogens they cover. The virus responsible for the 2003 outbreak of severe acute respiratory syndrome (SARS) is on many of the export control lists used around the world, but the lists do not refer to SARS-CoV or SARS-CoV-1. Instead, for example, both the US and UK export control lists and the Australia Group's list of human and animal pathogens and toxins for export control^12^ include “severe acute respiratory syndrome-related coronavirus.” The movement of viruses and their genes falling within this category requires export licenses. It is possible the authors of the export control lists deliberately chose to use vague language in order to capture a wider set of related viruses. However, because of this ambiguity, it is unclear whether export controls apply to SARS-CoV-2.

The term “severe acute respiratory syndrome-related coronavirus” is a classification used by the International Committee on Taxonomy of Viruses, which names viruses, to capture those that are closely related to SARS-CoV-1. In March 2020, the committee determined that SARS-CoV-2 was indeed a SARS-related coronavirus.^13^ As currently drafted, therefore, it appears to be covered by existing export controls.^†^ However, at a minimum, there is an urgent need for all governments that use the term “SARS-related coronavirus” to clarify whether export licenses are needed specifically for SARS-CoV-2.

The second reason export controls might apply is the sequence similarity (homology) between SARS-CoV-2 and previously sequenced SARS-CoV-1 isolates. Using the MUMmer algorithm via the JSpeciesWS online service,^14,15^ we see an 85% similarity between the recently sequenced Wuhan-Hu-1 SARS-CoV-2 isolate and the Tor2 SARS-CoV-1 patient isolate sequenced in Canada in 2017. This is in contrast to findings that members of the *Poxviridae* family (eg, goatpox and smallpox)—that are less than 55% identical—are both unambiguously on the list.^5^

The International Committee on Taxonomy of Viruses accepted that these viruses were sufficiently closely related to warrant categorizing them as the same species. When placing SARS-CoV-2 within its taxonomic tree, the group “recognizes this virus as forming a sister clade to the prototype human and bat severe acute respiratory syndrome coronaviruses (SARS-CoVs) of the species *Severe acute respiratory syndrome-related coronavirus*.”^13^ Perhaps this genetic similarity alone would be sufficient to capture SARS-CoV-2 under export controls. However, there is ambiguity about how much deviation from the reference sequence of a listed virus means that it is no longer covered.

Because export controls are meant to make it more difficult to access the agents that cause certain diseases, perhaps the term “SARS-related coronavirus” (as used in the export control lists) does not refer solely to a coronavirus genetically and taxonomically related to the one that causes SARS, but to ones that also cause diseases similar to SARS. If this is the case, SARS-CoV-2 would not need to be captured within this category, as COVID-19 is clinically different from SARS.

## Export Controls and SARS-CoV-2 Fragments

It is important to note that export controls—and the need to obtain a license when moving things internationally—do not apply only to whole viruses. As illustrated by changes made to the Australia Group rules in recent years, export controls also cover genetic elements and genetically modified organisms. This includes any gene or genes specific to any listed virus.^11^ If SARS-CoV-2 is covered by export controls, which it appears to be in countries that have not made any clarifications to the contrary, an export license may be required to move genetic material that contains its genes. That could include all synthetic DNA or RNA being used in critical research and development around the world. Imposing such licensing requirements could have a major impact on our efforts to detect, diagnose, survey, prevent, and treat this disease.

The possibility that genetic elements are included in export control measures poses many questions for consideration. What is meant by a gene here? Is it a specific sequence or a defined functional unit? How close to a reference sequence must a genetic element be to require an export license? Would a single-base substitution be sufficient to sidestep such a requirement? Would minor alterations to the sequence count even if they had no impact on the function of a gene? If a gene is a functional unit, then minor sequence variations that do not affect function are not relevant. If a gene has a specific sequence, then minor changes may affect the scope of coverage.

**Figure 1.**
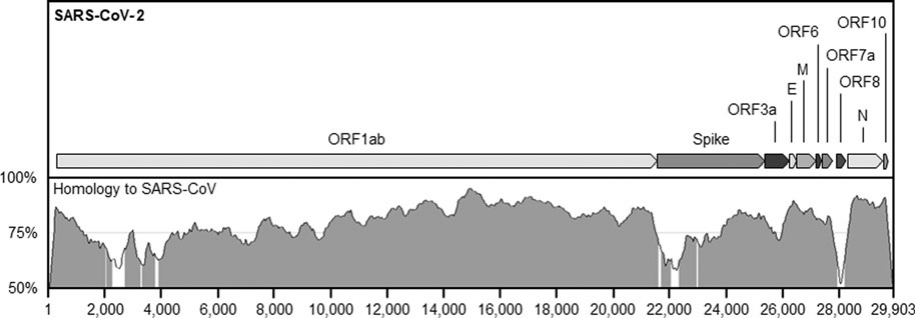
Sequence homology between the SARS-CoV-1 and SARS-SoV-2 viruses. Several portions of the SARS-CoV-2 genome show high levels of homology to the SARS-CoV genome. The top box shows the genome of SARS-CoV-2 with annotations of its protein-encoding open reading frames. The bottom box shows homology levels to SARS-CoV (Tor2 isolate) plotted using a sliding window of 100 bp. Regions where homology is over 70% within this window are shaded in gray. Homology levels were calculated using mVISTA. ORF, open reading frames; SARS-CoV, severe acute respiratory syndrome coronavirus.

In practice, there may be even more confusion. At least 1 peptide encoded by SARS-CoV-2 is nearly identical—over 95% sequence homology—to its counterpart in SARS-CoV-1. Several other proteins and genes are over 90% identical (Figure 1).^16-18^ Does this then mean that even if SARS-CoV-2 as a whole is not directly covered by export controls, many of its sequences are? Arguably, if that is the case, a researcher outside of the United States who wanted to export genetic material containing a gene from SARS-CoV-2 as part of the current pandemic response effort—and that gene is sufficiently similar to one in SARS-CoV-1—would then need a license. The answers to this and similar questions can have important practical implications. For instance, portions of the SARS-CoV-2 genome with high homology to other coronaviruses make good targets for diagnostic tests, as they are likely to be very similar in all patients. Diagnostic kits, therefore, routinely contain DNA fragments identical to these sequences, which are used as positive controls. Several kits currently in production target the N coding sequence and portions of the open reading frames ORF1ab coding sequence. Thus, it is conceivable that diagnostic kits could require export licenses.

In this context, it is critical to define what “specific to any listed virus” means. Does it apply only to unique genes—those that are found in no other organism? How does this work when 2 viruses are so closely related? How much deviation from the reference sequence of a listed virus counts as being specific? In some cases, the variation between certain genes in a listed and unlisted virus are only a small number of single point changes. What are the implications of highly conserved sequences present in both listed and unlisted relatives? How sure are we of the reference sequences? How do we deal with natural sequence variations within the same species?

## Preventing Future Confusion

Questions raised during the current pandemic highlight the need to improve our export control regimes. These improvements need not be radical and could be carried out in a phased manner.

To start, the lists of pathogens covered by export controls could be revised to ensure they make taxonomic sense. For example, the term “severe acute respiratory syndrome-related coronavirus” could be updated to better reflect the discovery of many closely related viruses that do not cause the physiological symptoms that led to SARS-CoV being included in export control lists.

A more ambitious short-term revision might list the diseases caused by pathogens rather than listing the pathogens. This would mean that any pathogen that causes SARS would be covered by export controls, but close relatives with a different clinical presentation (such as COVID-19) would not be. This approach could sidestep the need to define the genetic scope of the pathogens included on the list. Such an approach might also be useful for other pathogens, such as bacteria. For example, the US, UK, and Australia Group lists all include *Bacillus anthracis*, the causative pathogen of anthrax. Anthrax, however, can also be caused by a different bacterium (*Bacillus cereus* biovar *anthracis*).^19^ To capture this distinction, the United States added the other pathogen to its control list,^20^ but the United Kingdom and the Australia Group have not. Listing the diseases caused by pathogens, rather than listing the pathogens, would help clarify that any bacteria that cause anthrax require export licenses. Switching to a disease-based approach may, however, result in other challenges, such as a need to define which symptoms present in a given disease or how to know what disease an agent might cause without infecting a human or animal with it. In some cases, these challenges may be more manageable than attempting to define the sequence variations of a pathogen. For example, the World Health Organization already has an authoritative international listing of symptoms associated with a disease—the International Classification of Disease, which is currently in its 11th edition.^21^

If control lists remain focused on pathogens, resulting questions about which sequences are covered and the degree of sequence homology required, will ultimately need to be answered. As recently as 2010, this was considered to be over the horizon.^22^ However, it is a reasonable medium-term aim. In practice, answers to these questions are already being used by gene synthesis companies. They screen their orders, including against export control lists. To do this, the lists of controlled pathogens must be translated into a database of controlled sequences. These companies decide what to do with orders that are similar to controlled sequences and those that might have a similar biological function. They do this largely separately from the security expertise accessible to governments to identify unusual sequences of concern. Revising export controls to focus on sequences, rather than taxonomy, would allow for the creation of standardized databases for screening and accelerated development of democratized screening tools.^23^ Considerable thought has already gone into developing frameworks and systems in which such a database might be housed to maximize its use, while protecting against potential for misuse.^24,25^

Regardless of whether control lists are revised to focus on diseases or sequences, ultimately it is the biological function of the material that determines whether it might be misused to cause deliberate harm and whether it should be controlled. For example, anthrax was developed as a biological weapon in part because of the symptoms it causes and its environmental resistance—due to its production of a specific toxin and its ability to form spores, respectively. Both are biological functions. Efforts to determine what types of biological functions or which diseases are considered of concern, rather than attempting to identify all the responsible sequences, might avoid both the false positives and false negatives currently in evidence. Programs such as the Functional Genomic and Computational Assessment of Threats (Fun GCAT), and its successful progression to a second round in 2019, demonstrate progress in moving toward the ultimate goal of a function-based system.^26^

## Conclusion

During the current pandemic, there is a pressing need for clarity about which export licenses are required and for what. There is currently too much ambiguity about how export controls apply in our response to COVID-19. As discussed earlier, the current approach to export control lists is insufficient to address the realities of biological research. Confusion over whether export controls apply to the SARS-CoV-2 virus causing COVID-19 demonstrates that this lack of clarity can have very real public health consequences. Export controls that impede the ability to do research on SARS-CoV-2 in the midst of an ongoing pandemic are inappropriate and harmful, especially when samples of the virus are already widely available around the world. No controls should be placed—at least for now—on international transfers of SARS-CoV-2 samples or related genetic sequences. Any controls that capture such transfers should be relaxed, and if regulatory changes to that effect cannot be made quickly, export control authorities should clarify that enforcement discretion will be used to ensure that these exports in effect are not regulated. At a minimum, a fast track should be required for any license requests associated with SARS-CoV-2 or its fragments. Such an approach can always be revisited when the virus is not so widespread or the public health need is not as pressing. Proactive outreach to clearly explain the guidelines to those involved in relevant research and development is also required, as is connecting them to the appropriate decision-making processes.

Clarifying the intention of export controls to more closely align with their intended implementation is difficult but should not be a challenge from which we should shy away. Our governments rightly want to place barriers in the path of those who would use diseases to cause harm, but they must also avoid restricting critical public health efforts. We have a collective duty to improve the current guidelines to promote critical research during this global public health emergency.

## Notes

^*^ Because the Australia Group makes decisions by consensus, its member states would need to agree to such a statement. This would be time-consuming and further complicated by resource diversions and the global lockdown in place during the pandemic.

^†^ Interestingly, the United States continued to use the same phrase in an update of its export administration regulations published a week after the decision on the taxonomy of SARS-CoV-2. This reinforced the importance of the guidance issued at the end of February 2020 clarifying that this virus is not covered by export controls.
